# The Varicella-Zoster Virus ORF47 Kinase Interferes with Host Innate Immune Response by Inhibiting the Activation of IRF3

**DOI:** 10.1371/journal.pone.0016870

**Published:** 2011-02-09

**Authors:** Patricia Vandevenne, Marielle Lebrun, Nadia El Mjiyad, Isabelle Ote, Emmanuel Di Valentin, Yvette Habraken, Estelle Dortu, Jacques Piette, Catherine Sadzot-Delvaux

**Affiliations:** 1 GIGA-Research, Laboratory of Virology and Immunology, University of Liege, Liege, Belgium; 2 Laboratory of Molecular Oncology (LOM), Institut d'Investigació Biomèdica de Bellvitge (IDIBELL), Barcelona, Spain; 3 Department of Pathology, University of Liege, Liege, Belgium; University of Texas Medical Branch, United States of America

## Abstract

The innate immune response constitutes the first line of host defence that limits viral spread and plays an important role in the activation of adaptive immune response. Viral components are recognized by specific host pathogen recognition receptors triggering the activation of IRF3. IRF3, along with NF-κB, is a key regulator of IFN-β expression. Until now, the role of IRF3 in the activation of the innate immune response during Varicella-Zoster Virus (VZV) infection has been poorly studied. In this work, we demonstrated for the first time that VZV rapidly induces an atypical phosphorylation of IRF3 that is inhibitory since it prevents subsequent IRF3 homodimerization and induction of target genes. Using a mutant virus unable to express the viral kinase ORF47p, we demonstrated that (i) IRF3 slower-migrating form disappears; (ii) IRF3 is phosphorylated on serine 396 again and recovers the ability to form homodimers; (iii) amounts of IRF3 target genes such as IFN-β and ISG15 mRNA are greater than in cells infected with the wild-type virus; and (iv) IRF3 physically interacts with ORF47p. These data led us to hypothesize that the viral kinase ORF47p is involved in the atypical phosphorylation of IRF3 during VZV infection, which prevents its homodimerization and subsequent induction of target genes such as IFN-β and ISG15.

## Introduction

Innate immune response to viral infection involves the recognition of viral components through pathogen recognition receptors (PRRs) and the subsequent induction of type I IFNs (IFN-α/β) that can trigger the expression of antiviral proteins. Host PRRs recognize pathogen-associated molecular patterns (PAMPs) such as viral nucleic acids [1], [2]. PRRs comprise membrane-associated receptors such as Toll-like receptors (TLRs) and cytosolic receptors including RIG-I-Like Receptors (RLRs) and Nucleotide-binding Oligomerization Domain (NOD)-Like Receptors (NLRs) (for review [3]). More recently two new cytosolic receptors have been identified: the DNA-dependent Activator of IRFs (DAI), recognizing both microbial and host DNA [4], [5] and AIM2 (Absent in Melanoma 2) another DNA sensor [6], [7]. TLRs, RLRs and DAI are involved in viral detection and lead to the activation of several transcription factors including NF-κB, AP-1 and Interferon Regulatory Factor 3 (IRF3) that cooperate to induce the expression of IFN-β. In turn, the newly synthesized IFN-β, which is considered as the hallmark of the antiviral response, induces the expression of Interferon-Stimulated Genes (ISGs) encoding proteins such as 2′-5′ oligoadenylate synthase (OAS), dsRNA-dependent protein kinase R (PKR) and Interferon-Stimulated Gene 15 and 56 (ISG15 and ISG56) that are responsible for the establishment of an antiviral state in infected cells as well as in neighbouring non-infected cells [2], [8], [9]. By contrast to these PRRs, AIM2 induces the activation of the inflammasome, leading to the maturation of pro-IL-1β in mature IL-1β [4], [7], [10], [11].

In resting cells, IRF3 is present in a latent conformation in the cytoplasm. Upon viral infection, IRF3 is hyperphosphorylated on multiple serine and threonine residues located at the C-terminus [12], [13], [14]. Once phosphorylated, IRF3 homodimerizes and translocates into the nucleus where it associates with the co-activators CBP/p300 and activates the transcription of IFN-β in collaboration with NF-κB and AP-1 [13]. It has previously been demonstrated that the phosphorylation at the C-terminal domain of IRF3 is mediated by the two non-canonical IκB kinase (IKK)-related kinases IKK-ε and TBK1 [15], [16], [17], activated in response to the engagement of a PRR.

Varicella-Zoster Virus (VZV) is a human DNA virus belonging to the alphaherpesvirus subfamily. VZV is a neurotropic virus that causes two well-known pathologies: varicella (chicken pox) and herpes zoster (shingles). Varicella results from primary infection and is a common highly contagious childhood illness, associated with fever and generalized vesicular rashes [18]. Following the resolution of primary infection by the host immune system, VZV migrates along neuronal cell axon to reach dorsal root ganglia where it establishes a lifelong latent infection [19]. Reactivation from latency, due to a weakness of the immune system, leads to herpes zoster.

Varicella-Zoster Virus encodes two serine-threonine protein kinases highly conserved among herpesviruses, namely ORF47 protein (ORF47p) and ORF66 protein (ORF66p), both present in VZV virion. Heineman and colleagues previously showed that these two viral kinases are dispensable for viral replication in cell culture whereas others showed that they are essential for T cell tropism [20], [21], [22], [23], [24]. Moreover, ORF47p is essential for VZV replication in immature dendritic cells (DCs) but not in mature DCs [23]. ORF47p, which is homologous to HSV-1 UL13, hCMV UL97 and EBV BFLF4 protein kinases [25], [26], recognizes a consensus sequence similar to that of cellular casein kinase II (CKII) [27]. It has been published that ORF47p autophosphorylates *in vitro* and phosphorylates several viral proteins such as the major VZV transactivator IE62 [28], the glycoprotein gE, IE63 and the proteins encoded by ORF32 and ORF9 [29], [30], [31], [32]. However no cellular target has been identified so far.

On the contrary to ORF47p, that possesses homologs within α-, β- and γ-herpesvirus subfamilies, ORF66p is specific to the α-herpesvirus subfamily and is homologous to HSV-1 U_S_3 protein kinase. Eisfeld and colleagues previously showed that VZV ORF66p interacts with and phosphorylates the transcriptional regulatory protein IE62, affecting its subcellular distribution [33], [34]. ORF66p has been shown to protect VZV-infected cells from apoptosis by inhibiting Caspase 3 activation and to block IFN-γ-mediated STAT1 phosphorylation, leading to a decrease of MHCI expression at the surface of VZV-infected cells [20], [35].

Because IRF3 plays a major role in the type I IFN response to viral infection, many viruses have developed different strategies to subvert the host immune response by targeting this pathway. For example, Herpes simplex virus (HSV), Human cytomegalovirus (HCMV) and Epstein-Barr virus (EBV) encode proteins that interfere with the IRF3 pathway [36], [37], [38], [39], [40]. The activation of IRF3 in the context of VZV infection has been poorly studied although Sen and co-workers recently showed that the VZV immediate early protein IE62 inhibits its phosphorylation [41]. In the present study, we examined the activation of IRF3 in response to VZV and particularly the influence of the viral kinases encoded by the ORFs 47 and 66 on its activation. We showed that ORF47p but not ORR66p is involved in an atypical phosphorylation of IRF3 that is inhibitory since it prevents IRF3 homodimerization. Furthermore, we observed that mRNA amounts of two IRF3-dependent genes, IFN-β and ISG15, are increased in cells infected with a mutant virus unable to express the ORF47p (VZV ROka47S) compared to the wild-type (WT) virus. We also demonstrated that ORF47p interacts with IRF3. Based on these data, we hypothesized that VZV, through its kinase ORF47p, targets the IRF3 pathway. Therefore the viral kinase encoded by the ORF47 could play a role in mechanisms involved in immune evasion following infection with VZV.

## Results

### IRF3 is phosphorylated during VZV infection

Since IRF3 is a transcription factor playing a central role in the antiviral response, we decided to investigate whether IRF3 can be activated upon VZV infection. In resting cells IRF3 is present as two forms: form I and form II, corresponding respectively to the non-phosphorylated and the basally phosphorylated form of IRF3. These two forms appear as a doublet in SDS-PAGE. In general, viral-mediated phosphorylation of IRF3 occurs within the C-terminal domain on Ser385 and Ser386 residues as well as on the serine/threonine cluster located between amino acids 396 and 405 [42]. This hyperphosphorylation, in the context of an infection with the Sendai Virus, leads to the appearance of the forms III and IV that migrate more slowly on SDS-PAGE and can thus be discriminated from the forms I and II [14], [43], [44]. In the present study, we evaluated the ability of VZV infection to induce the appearance of such hyperphosphorylated forms of IRF3.

**Figure 1:**
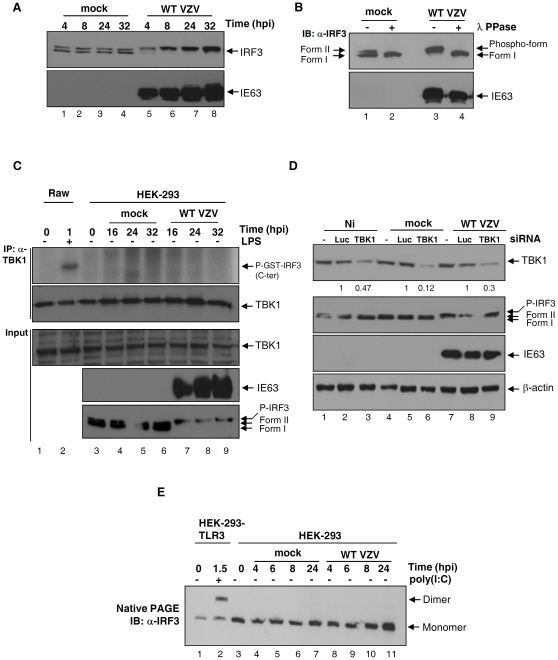
IRF3 is phosphorylated during VZV infection and then is unable to homodimerize. (A) HEK-293 cells were mock-infected or infected with the WT VZV during the indicated periods of time. Total cell lysates were harvested, resolved by SDS-PAGE and immunoblotted with specific antibodies against IRF3 and the viral protein IE63. (B) HEK-293 cells were mock-infected or infected with the WT VZV for 24 hours. Total cell lysates were performed, treated or not with the λ-phosphatase and then resolved by SDS-PAGE and immunoblotted with specific antibodies against IRF3 and IE63. (C) Raw 264.7 macrophages were stimulated or not with LPS for 60 min. HEK-293 cells were mock-infected or infected with the WT VZV during the indicated periods of time. Total cell extracts were performed and TBK1 was immunoprecipitated from these lysates with a specific antibody. The Immunocomplexes were subjected to an *in vitro* kinase assay in the presence of radiolabelled ATP and the substrate GST-IRF3 bearing the Carboxy- end of IRF3. Reactional mixes were then resolved by SDS-PAGE and TBK1 immunoprecipitation efficiency was analyzed by Western Blotting. Inputs were also resolved by SDS-PAGE and Western Blottings against TBK1, IE63 and IRF3 were performed (lower panel). (D) HEK-293 cells were transfected or not with 100 pmol of siRNAs directed against TBK1 or Firefly luciferase which was used as negative control. Cells were then non-infected, mock-infected or infected with the WT VZV and 24 hours later cells were harvested. IRF3 up-shift was monitored by SDS-PAGE. TBK1 knockdown was confirmed by Western Blot analysis using a specific antibody against TBK1. Specific antibodies against IE63 and β-actin were also used as control for infectivity and loading, respectively. (E) HEK-293 cells stably expressing TLR3 were stimulated or not with Poly (I:C) for 90 min. HEK-293 cells were mock-infected or infected with the WT VZV during the indicated periods of time. Total cell extracts were performed and IRF3 homodimerization was analyzed by native gel electrophoresis.

To analyze the phosphorylation of IRF3 in response to VZV infection, HEK-293 cells were either mock- or VZV-infected and collected various times post-infection. Total cell extracts were resolved by SDS-PAGE. In mock-infected cells, IRF3 appears as a doublet corresponding to the non-phosphorylated form (I) and the basally phosphorylated form (II) (Figure 1A, lanes 1–4). In VZV-infected cells a slower-migrating form of IRF3 appears as soon as 4 hours post-infection (hpi) (Fig. 1A, lane 5) and accumulates over time, being still present at 32 hpi (Figure 1A, lane 8). Western Blotting against the viral immediate-early protein IE63 was used to control the viral infection.

To verify whether the slower-migrating form of IRF3, which appears upon VZV infection, was effectively due to phosphorylation, total cell extracts both from mock- and VZV-infected cells were treated or not with λ-phosphatase during 45 min at 30°C. As expected, treatment of mock-infected cells with λ-phosphatase induces the disappearance of the form II compared to the untreated mock-infected cells (Figure 1B, lanes 1 & 2). In VZV-infected cells untreated with the enzyme, IRF3 appears up-shifted compared to untreated mock-infected cells (Figure 1B, lanes 1 & 3) and the treatment of infected cells with the λ-phosphatase totally brings down the slower-migrating form of IRF3 compared to untreated infected cells (Figure 1B, lanes 3 & 4). This result shows that the up-shift of IRF3 appearing upon VZV infection is caused by phosphorylation.

It has been previously demonstrated that the two non-canonical IκB kinase homologs, namely IκB kinase-ε (IKK-ε or IKKi) and TANK-binding kinase-1 (TBK1), are involved in the C-terminal phosphorylation of IRF3 upon dsRNA stimulation or viral infection [15], [16]. Since we have shown that IRF3 is phosphorylated in response to VZV, we wondered whether TBK1 was involved in VZV-mediated phosphorylation of IRF3. To answer this question, we have performed an *in vitro* Kinase Assay using TBK1 immunoprecipitated from HEK-293 cells infected or not with VZV and GST-IRF3 bearing the C-terminus of IRF3 as substrate. Raw macrophages treated with LPS were used as positive control (Figure 1C, upper panel, lane 2). Surprisingly, the TBK1 immunoprecipitated from VZV-infected cells is not able to phosphorylate the GST-IRF3 (Figure 1C, upper panel, lanes 7–9) whereas, in corresponding inputs, IRF3 slower-migrating form (referred to as P-IRF3 in the figure) is detectable (Figure 1C, lower panel, lanes 7–9). Since TBK1 is known to phosphorylate the serine 396 residue [16], TBK1 *in vitro* kinase assay result was confirmed by Western Blotting using an antibody that specifically recognizes the IRF3 phosphorylated on serine 396. According to the absence of TBK1 activation, no IRF3 phosphorylated on serine 396 has been detected in VZV-infected cells (data not shown). Furthermore, we demonstrated that, in cells in which TBK1 was knocked-down by specific siRNA, IRF3 is still phosphorylated and up-shifted in response to VZV infection (Figure 1D, lanes 7–9). The efficiency of TBK1 siRNA on the inhibition of the IRF3 pathway activation has been verified by analyzing the expression of IFN-β mRNA expression by semi-quantitative RT-PCR (data not shown). Collectively, our data suggest that VZV-dependent phosphorylation of IRF3 does not require TBK1 kinase activity. Therefore, we concluded that VZV induces the phosphorylation of IRF3 in a manner that appears to be TBK1-independent and we referred the VZV-mediated phosphorylation of IRF3 to as an atypical phosphorylation which is indicated as P-IRF3 in the figures. This observation also led us to hypothesize that this atypical phosphorylation may interfere with IRF3 activation.

C-terminal phosphorylation of IRF3 is a critical step for its activation since it allows its homodimerization and nuclear translocation. Since VZV infection leads to a TBK1-independent phosphorylation of IRF3, we wondered whether the VZV-mediated phosphorylation of IRF3 permits its homodimerization. HEK-293 cells were either mock-infected or VZV-infected and examined various times post-infection. IRF3 homodimerization was monitored by native PAGE as described previously [45]. HEK-293-TLR3 challenged with Poly (I:C) for 90 min were used as positive control (Figure 1E, lane 2). IRF3 homodimerization was observed neither in mock-infected cells (Figure 1E, lanes 4–7) nor in VZV-infected cells (Figure 1E, lanes 8–11). Taken together, these data suggest that VZV rapidly induces an atypical phosphorylation of IRF3 that is a non-activating phosphorylation since it does not allow IRF3 to homodimerize. Based on this, we suggest that the VZV-mediated phosphorylation of IRF3, which leads to its up-shift in SDS-PAGE, does not correspond to the activated hyperphosphorylated forms III and IV that are observed, for example, during an infection with the Sendai Virus [44].

### The VZV viral kinase encoded by the VZV ORF47 but not ORF66 is involved in the atypical phosphorylation of IRF3

**Figure 2:**
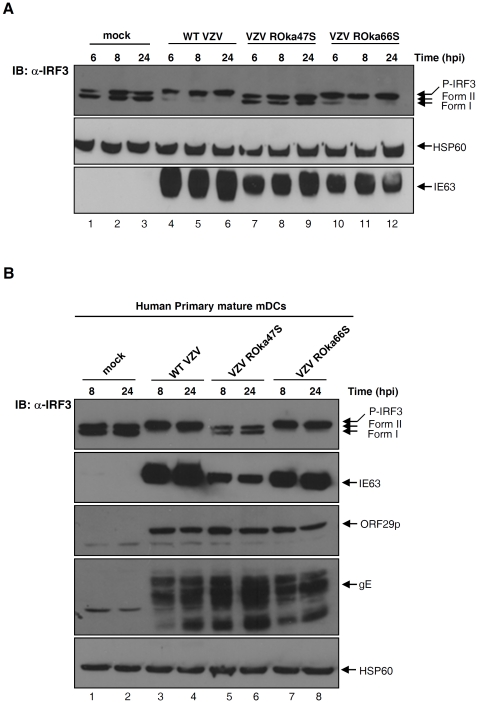
The VZV kinase ORF47p but not ORF66p is involved in the phosphorylation of IRF3. (A) HEK-293 cells were mock-infected or infected with the WT VZV and the two mutant viruses, namely VZV ROka47S and VZV ROka66S during the indicated periods of time. Total cell extracts were performed and IRF3 up-shift was monitored by SDS-PAGE. The membrane was immunoblotted with specific antibodies against IRF3, HSP60 and IE63. HSP60 and IE63 were used as control for loading and infectivity, respectively. (B) Mature mDCs were prepared as described in the Material and Methods and co-cultured with either non-infected MeWo cells or with MeWo cells infected either with the WT VZV, the mutant VZV Roka47S or Roka66S for 24 hours. Non-adherent mDCS were then collected and placed into fresh 6-well plates. Cells were harvested after supplemental 8 and 24 hours and total cell extracts were performed. The up-shift of IRF3 was monitored by SDS-PAGE. The membrane was immunoblotted with specific antibodies against IRF3 and HSP60. HSP60 was used as control for loading. Following viral proteins were also detected with specific antibodies: IE63, ORF29p and gE.

VZV expresses two kinases encoded by the ORF47 and ORF66. Some herpesviral kinases were reported to interfere with the activation of IRF3. For example, EBV is responsible for an inhibitory phosphorylation of IRF3 [40]. Therefore, we attempted to elucidate whether VZV kinases were implicated in the atypical phosphorylation of IRF3 observed following VZV infection. To answer this question, two mutant viruses unable to express either ORF47 (ROka47S) or ORF66 (ROka66S) proteins were used [21], [22]. HEK-293 cells were mock-infected or infected with the WT VZV, the VZV ROka47S and the VZV ROka66S. Cells were harvested at the indicated periods of time, total cell extracts were performed and IRF3 up-shift was assessed by SDS-PAGE. As already shown above, IRF3 is not up-shifted in mock-infected cells (Figure 2A, lanes 1–3) whereas WT VZV rapidly leads to the appearance of the IRF3 phosphorylated form (Figure 2A, lanes 4–6). Surprisingly, IRF3 is not up-shifted when cells are infected with the mutant ROka47S (Figure 2A, lanes 7–9) while phosphorylated form of IRF3 is still observed in cells infected with the mutant ROka66S (Figure 2A, lanes 10–12) as observed with the WT virus. This result indicates that the viral kinase ORF47p but not ORF66p is responsible for the appearance of the slower-migrating form of IRF3 during VZV infection.

To confirm the relevance of the role of ORF47p in the phosphorylation of IRF3 during VZV infection, the phosphorylation of IRF3 was evaluated in human primary mature myeloid dendritic cells (mDCs) infected with the different viruses. mDCs were prepared from human peripheral blood and infected with the WT VZV or with the two mutant viruses (ROka47S and ROka66S). Eight and 24 hours post-infection, mDCs were collected, total cell extracts were performed and IRF3 up-shift was analyzed by SDS-PAGE. IRF3 is not up-shifted in mock-infected mDCs (Figure 2B, lanes 1 & 2) while infection with the WT VZV leads to the appearance of an IRF3 slower-migrating form (Figure 2B, lanes 3 & 4). As expected, IRF3 is no more up-shifted in mDCs infected with the mutant VZV ROka47S (Figure 2B, lanes 5 & 6) while it is still up-shifted in mDCs infected with the mutant VZV ROka66S (Figure 2B, lanes 7 & 8). The ability of the DCs to support the replication was verified by analysis of VZV proteins from the three classes of genes, namely IE63, ORF29p and gE (Figure 2B, lower panels). This result confirms the observations in HEK-293 cells, highlighting that ORF47p is involved in the atypical phosphorylation of IRF3 during VZV infection in primary cells. Taken together, these data provide evidences that the viral kinase ORF47p, but not ORF66p, plays an important role in the phosphorylation of IRF3 following infection with VZV. Our experiments were then focused on the effects of the viral kinase ORF47p rather than ORF66p on the activation of IRF3 during VZV infection.

VZV ROka47S and ROka66S are derived from the VZV vaccine strain ROka. Since we established comparisons between the WT VZV, the vaccine ROka47S and ROka66S, we have verified, by SDS-PAGE, that both the WT and the vaccine strains behave the same in term of IRF3 phosphorylation (data not shown).

### Expression of exogenous ORF47p restored the up-shift of IRF3 in cells infected with the mutant virus ROka47S

**Figure 3:**
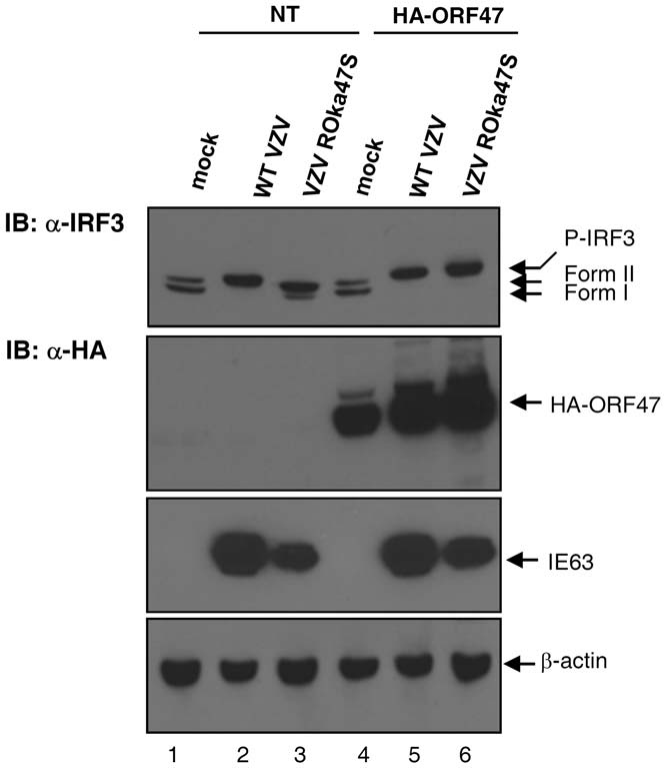
Expression of exogenous ORF47p restores the phosphorylation of IRF3 in cells infected with VZV ROka47S. HEK-293 cells were transfected or not with HA-ORF47 expressing vector. 24 hours later, cells were mock-infected or infected with either the WT VZV or the mutant VZV ROka47S for another 24 hours. Total cell extracts were performed, resolved by SDS-PAGE and immunoblotted with specific antibodies against IRF3, HA-tag, IE63 and β-actin. Anti-HA antibody was used to control the transfection efficiency. IE63 was used as a control for infectivity while β-actin was used as a loading control.

To objectivate in another way the role of the viral kinase ORF47p in the atypical phosphorylation of IRF3 during VZV infection, we decided to rescue the expression of ORF47p in HEK-293 cells infected or not with the mutant VZV ROka47S. HEK-293 cells were transfected or not with HA-ORF47 expressing vector. Twenty four hours later, cells were mock-infected or infected with either the WT VZV or the mutant VZV ROka47S for 24 hours. Cells were then harvested and total cell lysates were performed. IRF3 up-shift was monitored by SDS-PAGE. Infection with the WT VZV leads to the up-shift of IRF3 (Figure 3, lane 2) whereas no up-shift is observed in cells infected with VZV ROka47S (Figure 3, lane 3). The restoration of ORF47p expression in cells infected with VZV ROka47S leads to a recovery of the shift showing a migration profile similar to the one observed in cells infected with the WT VZV (Figure 3, lanes 2 & 6). Importantly, the ORF47p expression in mock-infected cells does not induce the up-shift of IRF3 (Figure 3, lane 4) suggesting that, alone, ORF47p is unable to induce the phosphorylated form of IRF3 that is up-shifted in SDS-PAGE.

### In absence of ORF47p expression, the osphorylation of IRF3 on serine 396 is restored and IRF3 can homodimerizeph

As already mentioned above, TBK1 was previously reported to phosphorylate the C-terminal cluster of IRF3 that leads to its homodimerization and its subsequent nuclear translocation [15]. The C-terminal cluster that is phosphorylated by TBK1 comprises several serine and threonine residues including the serine 396 which phosphorylation is a prerequisite for IRF3 homodimerization [12], [48].

**Figure 4:**
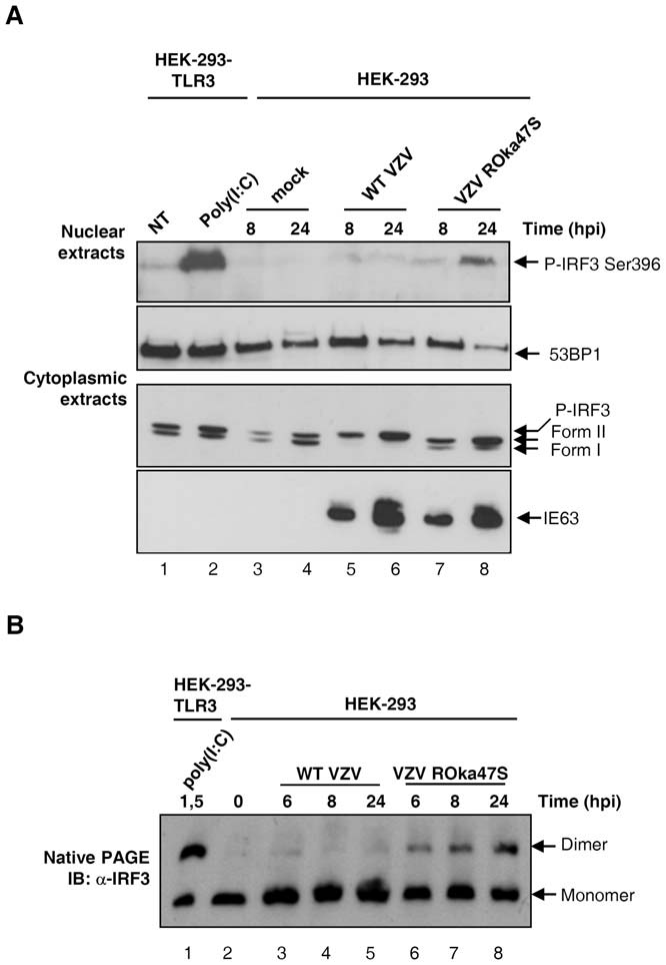
In absence of ORF47p expression, IRF3 is phosphorylated on serine 396 and homodimerizes again. HEK-293 cells stably expressing TLR3 were stimulated or not with Poly (I:C) for 90 min. HEK-293 cells were mock-infected or infected with either the WT VZV or the mutant VZV ROka47S during the indicated periods of time. (A) Cytoplasmic and nuclear extracts were harvested. Nuclear proteins were resolved by SDS-PAGE and immunoblotted with specific antibodies against IRF3 phosphorylated on serine 396 and the nuclear protein 53BP1 while cytoplasmic proteins were used to perform Western Blotting against IRF3 and the viral protein IE63 using specific antibodies. (B) Total cell extracts were performed and IRF3 homodimerization was analyzed by native gel electrophoresis. The membrane was then immunoblotted with a specific antibody against IRF3.

As demonstrated above, VZV induces an atypical phosphorylation of IRF3 that is TBK1-independent and is a non-activating phosphorylation depending on the presence of the viral kinase ORF47p. Based on this, we wondered whether the phosphorylation of IRF3 on serine 396 could be induced in cells infected with a mutant virus unable to express the viral kinase ORF47p. HEK-293 cells were mock-infected, infected with either the WT VZV or the mutant VZV ROka47S. HEK-293 cells stably expressing TLR3 treated with Poly (I:C) were used as a positive control. Cytoplasmic and nuclear extracts were harvested at the indicated times. Nuclear proteins were resolved by SDS-PAGE and Western Blotting against IRF3 phosphorylated on serine 396 was performed using a phosphospecific antibody (Figure 4A). As expected, Poly (I:C) induces the phosphorylation of IRF3 on serine 396 (Figure 4A, upper panel, lane 2) whereas no phosphorylation on this residue is detected neither in mock-infected cells (Figure A4, upper panel, lanes 3 & 4) nor in VZV-infected cells (Figure 4A, upper panel, lanes 5 & 6) while the phosphorylation of IRF3 on serine 396 seems to be recovered in cells infected with the mutant VZV ROka47S (Figure 4A, upper panel, lane 8). Western Blotting against an exclusively nuclear protein 53BP1 was used as a control for nuclear proteins (Figure 4A, lower panel). Corresponding cytoplasmic extracts were also resolved in SDS-PAGE to verify the IRF3 up-shift (Figure 4A, lower panel). As demonstrated above, IRF3 is up-shifted in cells infected with the WT VZV (Figure 4A, lower panel, lanes 5 & 6) whereas no up-shift is detected in cells infected with the mutant VZV ROka47S (Figure 4A, lower panel, lanes 7 & 8). Western Blotting against the viral protein IE63 was also performed to control cell infectivity.

Although IRF3 is phosphorylated on serine 396 in response to Poly (I:C) treatment as well as in cells infected with the mutant VZV ROka47S (Figure 4A, upper panel, lanes 2 & 8) it is not up-shifted in SDS-PAGE (Figure 4A, lower panel, lanes 2 & 8). This result demonstrates that the phosphorylation of IRF3 on serine 396 that leads to its activation is not necessarily accompanied with the appearance, in SDS-PAGE, of up-shifted forms of IRF3 as it can be observed, for example, during Sendai Virus infection [44].

Since phosphorylation on serine 396 seems to be restored in cells infected with the mutant VZV ROka47S, we wondered whether IRF3 could also homodimerize in absence of ORF47p expression. HEK-293 cells were mock-infected or infected with either the WT VZV or the mutant VZV ROka47S. Cells were harvested at various times post-infection and IRF3 homodimerization was monitored by native PAGE. Poly (I:C) treatment, known to induce the homodimerization of IRF3, was used as a positive control in HEK-293-TLR3 (Figure 4B, lane 1). As expected, IRF3 does not homodimerize in VZV-infected cells (Figure 4B, lanes 3-5) but interestingly, IRF3 homodimers are present in cells infected with the mutant VZV ROka47S (Figure 4B, lanes 6-8). This result is in accordance with our hypothesis that the viral kinase ORF47p interferes with the activation of IRF3 during VZV infection. VZV, through its ORF47p kinase, induces an atypical phosphorylation of IRF3 that does not occur on serine 396 and prevents its homodimerization. Altogether, our data suggest that the modified form of IRF3, induced upon VZV infection that leads to its upshift in SDS-PAGE, is indeed due to some phosphorylation of IRF3 but has to be distinguished from the activated hyperphosphorylated forms III and IV.

### IRF3-dependent gene expression is increased in cells infected with VZV ROka47S compared to WT VZV

**Figure 5:**
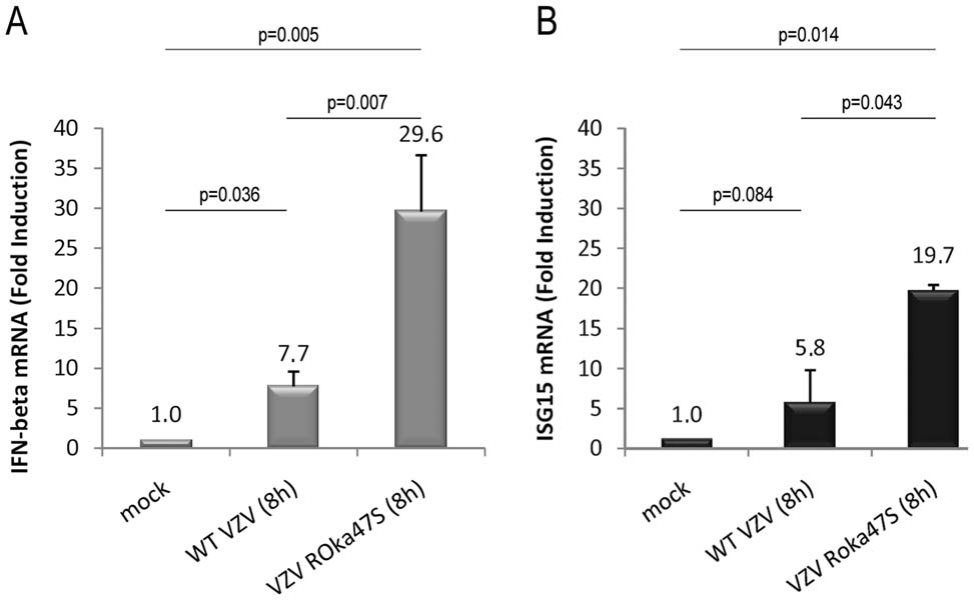
In absence of ORF47p expression, IRF3-dependent gene expression is increased. HEK-293 cells stably expressing TLR3 were mock-infected or infected with either the WT VZV or the mutant VZV ROka47S for 8 hours. Total RNA were harvested and subjected to a Reverse Transcription reaction. cDNA were subjected to a quantitative Real Time PCR in presence of SYBR Green. Primers directed to *ifnβ* (A) and *isg15* (B) were used. RT-PCR was normalized using the 18S RNA expression level. Error bars indicate the standard deviation of the mean.

Since IRF3 is able to form homodimers in response to VZV infection when the viral kinase ORF47p is not expressed, we wondered whether infection with the mutant VZV ROka47S leads to an increase of IRF3-dependent gene expression compared to the WT VZV infection. To answer this question, the expression of two IRF3 target genes, namely IFN-β and ISG15 was analyzed by qRT-PCR in HEK-293 cells stably expressing TLR3. HEK-293 cells stably expressing TLR3 were mock-infected or infected with either the WT VZV or the mutant VZV ROka47S for 8 h. Cells were harvested and total RNA were isolated and subjected to a quantitative RT-PCR in presence of SYBR Green. As shown in Figure 5A, infection with the WT VZV slightly increases the expression of IFN-β whereas infection with VZV ROka47S leads to a much higher expression of IFN-β mRNA. Similarly, the same profile is observed with ISG15 mRNA (Figure 5B). Altogether, these data revealed that, during infection with the WT VZV, the viral kinase ORF47p is responsible for the decrease of IRF3-dependent genes expression.

### ORF47p interacts with IRF3

**Figure 6:**
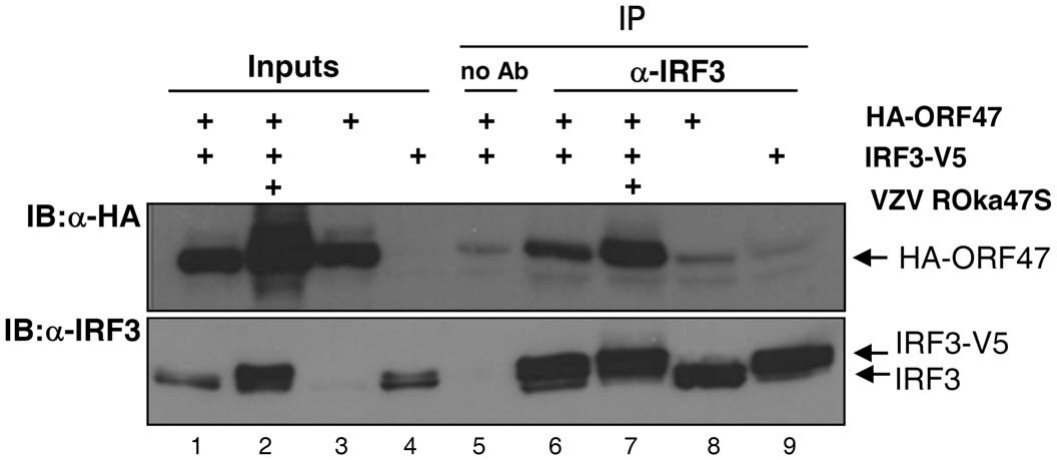
IRF3 physically interacts with ORF47p. HEK-293 cells were transfected either with HA-tagged ORF47 or V5-tagged IRF3 or co-transfected with both plasmids. 24 hours later, cells were infected or not with the mutant VZV ROka47S for another 24 hours. Either IRF3 or HA-ORF47 was immunoprecipitated from total extracts with anti-IRF3 and anti-HA antibodies. Inputs and immune complexes were then analyzed by Western Blotting using specific antibodies against HA-tag and IRF3. A negative control without antibody was also performed.

Next, we decided to determine, via co-immunoprecipitation, if ORF47p and IRF3 can physically interact. HEK-293 cells were transfected either with HA-tagged ORF47 (Figure 6, lanes 3 & 8) or V5-tagged IRF3 (Figure 6, lanes 4 & 9) or co-transfected with both plasmids (Figure 6, lanes 1, 2, 5, 6 & 7). Twenty four hours later, cells were infected or not with VZV ROka47S for another 24 hours. Inputs and immune complexes were analyzed by Western Blotting. Immunoprecipitation efficiency was checked (Figure 6, lower panel, lanes 6–9) and a negative control without antibody was performed (Figure 6, lane 5). We showed that, when immunoprecipitated, IRF3 efficiently co-precipitates HA-ORF47 even in absence of viral infection (Figure 6, lanes 6 & 7). Similarly, when immunoprecipitated, HA-ORF47p co-precipitates IRF3 (data not shown).

### The viral kinase ORF47p is not responsible for the VZV-mediated inhibition of NF-κB activation

**Figure 7:**
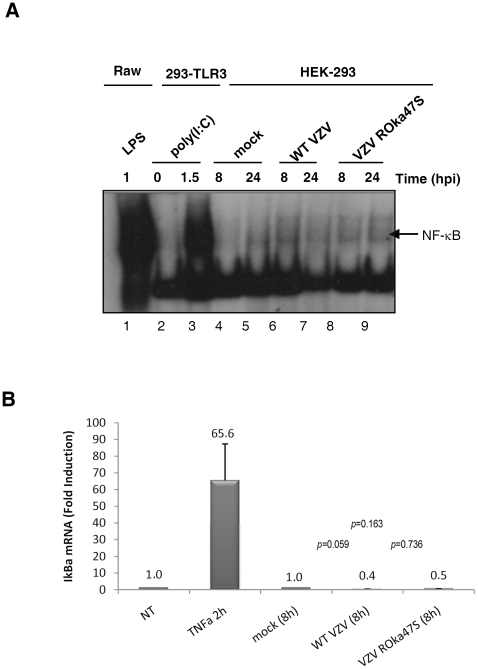
ORF47p is not responsible for the inhibition of NF-κB pathway during VZV infection. (A) Raw 264.7 macrophages were stimulated with LPS for 60 min. HEK-293 cells stably expressing TLR3 were stimulated or not with Poly (I:C) for 90 min. HEK-293 cells were mock-infected or infected with either the WT VZV or the mutant VZV ROka47S during the indicated periods of time. Various times post-infection, cells were collected and nuclear extracts were performed. 5 µg of nuclear proteins were used to study the NF-κB binding activity by EMSA using a radiolabeled probe carrying the NF-κB consensus sequence of HIV LTR promoter. (B) HEK-293 cells stably expressing TLR3 were stimulated or not with TNFα for 2 hours. Cells were mock-infected or infected with either the WT VZV or the mutant VZV ROka47S for 8 hours. Total RNA were harvested and subjected to a Reverse Transcription reaction. cDNA were subjected to a quantitative Real Time PCR in presence of SYBR Green and primers directed to *iκbα*. RT-PCR was normalized using the 18S RNA expression level. Error bars indicate the standard deviation of the mean.

Our data showed that VZV targets the activation of IRF3 by inducing its phosphorylation that is dependent on the viral kinase ORF47p. This phosphorylation inhibits IRF3 homodimerization preventing the subsequent induction of target genes such as IFN-β and ISG15, as demonstrated by qRT-PCR experiments. The induction of IFN-β expression requires the cooperation between IRF3 and NF-κB. We and others have recently demonstrated that VZV interferes with the activation of NF-κB [49], [50]. As shown above, we observed that IFN-β expression is very low following infection with VZV. Therefore, we wondered whether this low level of expression was also due to an interference with the activation of NF-κB and whether the activation of NF-κB is restored following infection with the mutant VZV ROka47S. In order to investigate whether the viral kinase ORF47p is involved in this VZV-mediated inhibition of NF-κB activation, the NF-κB binding activity was examined by electrophoretic mobility shift assay (EMSA). Raw macrophages were treated with LPS for one hour and HEK-293 cells stably expressing TLR3 were left untreated or treated with Poly (I:C) for 90 min. HEK-293 were mock-infected or infected with either the WT VZV or the mutant ROka47S. Cells were harvested at the indicated periods of time and nuclear extracts were performed. Nuclear extracts were then analyzed by electrophoretic mobility shift assay using a probe carrying the κB consensus sequence of the HIV LTR promoter. As shown in Figure 7A, treatment with LPS and Poly (I:C) induce a NF-κB DNA binding on the probe (Figure 7A, lanes 1 & 3). As expected, no binding is induced both in mock-infected cells (Figure 7A, lanes 4 & 5) and in cells infected with the WT VZV (Figure 7A, lanes 6 & 7). Furthermore, NF-κB DNA binding is not restored in cells infected with the mutant VZV ROka47S (Figure 7A, lanes 8 & 9). In order to confirm this result, the expression of IκBα mRNA, a NF-κB inducible gene, was carried out by qRT-PCR (Figure 7B). HEK-293 cells stably expressing TLR3 were left untreated or treated with TNFα (500 U/ml) for 2 hours. Cells were mock-infected or infected with either the WT VZV or the mutant VZV ROka47S for 8 hours. Cells were harvested and total RNA were isolated and subjected to a quantitative RT-PCR. As shown in the Figure 7B, the treatment with TNFα induces the expression of IκBα mRNA whereas the amount of IκBα mRNA is not increased neither in cells infected with the WT VZV nor in cells infected with the mutant VZV ROka47S. Therefore, these data are in accordance with the mobility shift assay experiment and collectively, they suggest that the activation of NF-κB is not modulated by the viral kinase ORF47p unlike IRF3. Thereby, these observations led us to hypothesize that the viral kinase ORF47p only targets the IRF3 pathway to prevent subsequent induction of IFN-β.

## Discussion

The innate immune response constitutes the first line of defence and plays an important role in the detection of invading pathogens therefore limiting the spread of infectious agents. The innate immune response is characterized particularly by the activation of IRF3 which plays a central role in the expression of IFN-β which in turn, induces the expression of interferon stimulated genes (ISGs) encoding proteins playing important roles in mounting an antiviral response leading to the clearance of pathogens. Viruses are considered as obligate parasites that use host machinery to replicate. Therefore, viruses have developed mechanisms to subvert host immune responses in order to increase viral spread. Herpesviruses are characterized by the establishment of a lifelong latency in host after primary infection. Thus, they developed various strategies to evade and bypass host innate immune responses. Indeed, it is well documented that Herpesviruses such as Herpes simplex virus type 1 (HSV-1), human Cytomegalovirus (hCMV) and Epstein-Barr virus (EBV) encode proteins that interfere with host innate immune responses and more particularly with the IRF3 pathway. For example, HSV-1 ICP0 and hCMV IE86 were reported to interfere with IRF3 activation and/or IFN-β expression [37], [51], [52]. In addition, some viral kinases were recently reported to interfere with the IRF3 activation [40], [46], [47]. In this study, we have attempted to investigate whether VZV interferes with the IRF3 pathway and whether the two VZV kinases, ORF47p and ORF66p, are involved in this VZV-mediated interference. The activation of IRF3 pathway during VZV infection was carried out in HEK-293 cells and the main results presented in this work can be summarized as follow: (i) Infection with VZV induces a rapid and persistent phosphorylation of IRF3 that does not require TBK1 kinase activity; (ii) IRF3 does not homodimerize upon infection with VZV; (iii) The viral kinase ORF47p but not ORF66p is involved in the VZV-mediated phosphorylation of IRF3 in HEK-293 cell line but also in human primary dendritic cells; (iv) When ORF47p expression is restored in cells infected with the mutant ROka47S, IRF3 is up-shifted again. (v) In absence of ORF47p expression, IRF3 phosphorylation on serine 396 and IRF3 homodimerization are restored whereas NF-κB activation is not restored; (vi) IFN-β and ISG15 mRNA amounts are increased in absence of ORF47p expression; (vii) Exogenous HA-ORF47p physically interacts with IRF3 in transient transfection.

To be activated, IRF3 requires phosphorylation at key serine/threonine residues located at the C-terminus of the protein. Among these residues, serine 396 was identified as the minimal phosphoacceptor site required for *in vivo* activation of IRF3 [12]. Since IRF3 is a transcription factor playing a central role in the activation of innate immune response, viruses often inhibit its activation and/or subsequent induction of target genes such as IFN-β. Here, we showed for the first time that, following infection with VZV, an IRF3 slower-migrating form appears as soon as 4 hours post-infection and accumulates over time. Morevover, we highlighted that this slower-migrating form is caused by phosphorylation as shown by λ-phosphatase treatment. TBK1 is known to phosphorylate IRF3 at the C-terminus leading to its homodimerization and its subsequent nuclear translocation [15], [16]. Here, we demonstrated, in an *in vitro* assay, that TBK1 is not activated in VZV-infected cells as, once immunoprecipitated, it is unable to phosphorylate the GST-IRF3 bearing the Carboxy-end of the protein. This result was confirmed by Western Blotting using an antibody that specifically recognizes the IRF3 phosphorylated on serine 396. Moreover, we showed, by using specific siRNA against TBK1, that this cellular kinase does not seem to be required for the VZV-dependent phosphorylation of IRF3 since the partial depletion of TBK1 does not impact on the up-shift observed. Therefore, we concluded that VZV induces the phosphorylation of IRF3 in a manner that appears to be TBK1-independent and we referred the VZV-mediated phosphorylation of IRF3 to as an atypical phosphorylation. Furthermore, this observation led to the hypothesis that this atypical phosphorylation may interfere with IRF3 activation. We verified this hypothesis by analyzing the homodimerization of IRF3 by native PAGE and surprisingly, our result demonstrated that the VZV-induced phosphorylation of IRF3 does not lead to the formation of IRF3 homodimers.

Altogether, our data led us to conclude that VZV infection induces an atypical phosphorylation of IRF3, which leads to the appearance of an up-shifted form in SDS-PAGE but not to the homodimerization of the protein and which does not concern the critical residue serine 396 and therefore has to be distinguished from the activating hyperphosphorylation leading to the so-called forms III and IV. Therefore, like other members of its family, VZV is able to interfere with the activation of IRF3 following viral infection. Indeed, infections with HSV-1 and hCMV rapidly activate IRF3 following virus binding and entry [53], [54], [55]. However, these two herpesviruses encode proteins that antagonize IRF3 activation and subsequent type I IFNs expression [37], [51], [52], [56], [57], [58]. On the contrary to HSV-1 and hCMV, our data demonstrated that VZV rapidly prevents IRF3 from homodimerization as soon as 4 hours post-infection and probably does not require *de novo* synthesis of viral proteins. Therefore, we hypothesized that one or several tegument proteins released into the cytoplasm soon after viral entry could rapidly encounter IRF3 and induce its atypical phosphorylation preventing thereby its homodimerization. VZV tegument proteins include the major viral transactivator, IE62 as well as IE63, ORF9p and the two viral kinases, namely ORF47p and ORF66p. Based on this, we decided to analyze whether the two VZV kinases, namely ORF47p and ORF66p are involved in the VZV-mediated phosphorylation of IRF3. Using two mutant viruses unable to express either ORF47p (ROka47S) or ORF66p (ROka66S), we demonstrated for the first time that, during viral infection, the kinase ORF47p but not ORF66p is involved in the VZV-mediated phosphorylation of IRF3 since it is no more up-shifted in absence of ORF47p as shown by Western Blotting. We have confirmed this finding in human primary dendritic cells highlighting the relevance of ORF47p in the phosphorylation of IRF3 following VZV infection. Furthermore, by restoring the expression of ORF47p in cells infected with the mutant VZV ROka47S, we showed that IRF3 recovers a migration profile similar to that observed in cells infected with the WT virus. However, we observed that, alone, ORF47p is unable to induce the up-shift of IRF3 suggesting that ORF47p may need other VZV protein(s) to induce IRF3 phosphorylation during VZV infection.

Furthermore, we have shown that, in absence of ORF47p expression, the phosphorylation of IRF3 on serine 396 is restored and IRF3 can homodimerize again. So, it appears that the atypical phosphorylation of IRF3, which depends on the viral kinase ORF47p, prevents IRF3 classical phosphorylation on serine 396 and subsequent IRF3 homodimerization. Accordingly, we showed that, in absence of ORF47p expression, mRNA amounts of two IRF3 target genes, namely IFN-β and ISG15, are significantly increased in cells infected with the mutant ROka47S compared to cells infected with the WT VZV. Importantly, our data are in line with those of Wang and colleagues showing that the EBV viral kinase BGLF4, homologous to ORF47p, inhibits the activation of IRF3 [40]. But, on the contrary to ORF47p, it was reported that BGLF4-mediated phosphorylation of IRF3 does not prevent its homodimerization and subsequent nuclear translocation. In addition, other herpesviral kinases such as HSV-1 UL13 kinase and the murine MHV-68 ORF36 kinase were reported to interfere with type I IFNs expression and with IRF3 binding to CBP/p300, respectively [46], [47]. EBV BGLF4, HSV-1 UL13, MHV-68 ORF36 and VZV ORF47p are homologous to each other and are conserved kinases among Herpesviruses in both human and mice [59] showing their importance in evasion of host innate immune response as well as the relevance of ORF47p in the inhibition of IRF3 activation during VZV infection. However, the complete mechanism by which ORF47p induces the phosphorylation of IRF3 is still to be investigated. Indeed, we do not know whether ORF47p directly phosphorylates IRF3 and whether the kinase activity of ORF47p is required for this function. We also do not know the region of IRF3 that is targeted in the presence of ORF47p. ORF47p has been previously reported to recognize a consensus sequence similar to that of the cellular CKII [27] and the *in silico* analysis of IRF3, using the Scansite software, reveals the presence of four potential CKII-like sites in the N-terminal region of IRF3. Therefore, we intend to investigate whether these predicted sites are targeted by the ORF47p. Collectively, our data suggest that VZV interferes with the activation of IRF3 through its kinase ORF47p, which induces an atypical phosphorylation of IRF3 that does not allow the classical phosphorylation on serine 396 and subsequent IRF3 homodimerization. Hence, infection with the mutant virus unable to express the viral kinase ORF47p results in the restoration of IRF3 serine 396 phosphorylation, homodimerization and an enhanced expression of IRF3 target genes such as IFN-β and ISG15.

Next, we showed, by co-immunoprecipitation in overexpressing cells, that IRF3 physically interacts with ORF47p. As ORF47p, alone, is able to interact with IRF3 but unable to induce its up-shift, we can postulate that another VZV protein is needed to allow IRF3 phosphorylation by ORF47p. This other VZV protein should also be a tegument protein since VZV-mediated IRF3 phosphorylation happens very early after infection. Very recently, Sen and co-workers demonstrated that the major VZV transactivator, IE62, interferes with the C-terminal phosphorylation of IRF3 thereby preventing IFN-β expression in absence of synthesis of *de novo* viral proteins [41]. IE62, along with ORF47p, is a tegument protein and both are consequently released in the cytoplasm of host cell upon viral entry. Since IE62 has been reported to be a substrate for the VZV kinase ORF47p [32], we can postulate that IE62 is present in the complex composed of IRF3 and ORF47p and could induce a conformational change thereby permitting the ORF47p-mediated phosphorylation of IRF3. We can also postulate that, in absence of the kinase ORF47p, IE62 is not phosphorylated anymore and may prevent the VZV-mediated inhibition of IRF3 as it was previously suggested for HSV-1 UL13 kinase and its substrate, ICP0 [46]. Finally, it is possible that IE62 and ORF47p act at different levels of the IRF3 pathway leading *in fine* to its inhibition.

The only common conclusion we can draw in comparison with the work of Sen and co-workers is the inhibition of the IRF3 pathway following infection with VZV. One should notice that Sen and colleagues showed that IE62 inhibits IRF3 phosphorylation only by transient transfection experiments using artificial expression of TBK1, IRF3 and IE62, whereas our data have been conducted in a more physiologically relevant context. Indeed, the main experiments demonstrating that ORF47p interferes with the activation of IRF3 were performed in the context of viral infection using WT VZV, vaccine ROka and mutant strains. Moreover, the activation of IRF3 was evaluated on the endogenous protein by Western Blotting. As IE62 is a potent transactivator essential for the viral replication, it is not easy to investigate its potential role in the inhibition of IRF3 in VZV-infected cells. Nevertheless, as IE62 is a target of ORF47p, it would be interesting to explore the possibility that they both play a role in the VZV-induced inhibitory phosphorylation of IRF3.

As already mentioned above, we observed that VZV interferes with the activation of IRF3 and the subsequent induction of two IRF3-dependent genes, namely IFN-β and ISG15 through its kinase ORF47p. We and other have previously shown that NF-κB activation was inhibited during VZV infection [49], [50]. Since IFN-β expression is induced by the cooperation between IRF3 and NF-κB, we wondered whether the decrease of IFN-β expression during VZV infection was also due to an inhibition of NF-κB activation. To answer this question, we investigated whether the absence of ORF47p expression restores the activation of NF-κB following infection with VZV. Our data showed that the activation of NF-κB is not restored upon infection with the mutant virus that does not express the ORF47p (ROka47S) compared to infection with the WT virus. Considering this finding, we suggest that the decrease of IFN-β expression during VZV infection is due to an ORF47p-mediated inhibition of IRF3 activation but not NF-κB.

**Figure 8:**
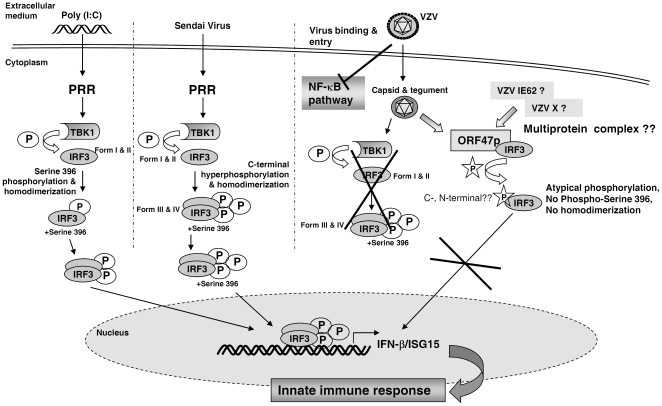
Model of VZV-mediated interference with IRF3 activation. Upon VZV entry, the viral kinase ORF47p, which is part of the VZV tegument, is released in the cytoplasm where it can encounter IRF3 and induce its atypical phosphorylation leading to an up-shift in the electrophoretic mobility of IRF3. Our data demonstrated that VZV does not activate the cellular kinase TBK1 and that VZV ORF47p-mediated phosphorylation of IRF3 does not occur on the classical serine 396. Furthermore, this atypical phosphorylation does not allow IRF3 homodimerization and subsequent induction of target genes such as IFN-β and ISG15. Consequently, the phosphorylated forms of IRF3 engendered by VZV through its ORF47 kinase, even if leading to an up-shift in SDS-PAGE, have to be distinguished from the hyperphosphorylated forms III and IV induced by other viruses such as the Sendai Virus. In addition, we have shown, in cells infected with a mutant virus unable to express the viral kinase ORF47, that the phosphorylation of IRF3 on serine 396 is restored. Hence, IRF3 homodimerizes again in cells infected with this ORF47 deficient virus. However, we failed to detect an up-shift of IRF3 in SDS-PAGE in these infected cells. Surprisingly, IRF3 showed the same behaviour in response to Poly (I:C) treatment suggesting that IRF3 phosphorylation on serine 396 and subsequent homodimerization are not necessarily accompanied with an up-shift in SDS-PAGE as observed in the context of an infection with the Sendai Virus. This also suggests that another activated form of IRF3, different than the forms III and IV, can exist. Until now, we still do not know whether ORF47p requires other viral proteins such as IE62 to mediate IRF3 phosphorylation following VZV infection. We also do not know whether this phosphorylation occurs at the C- or N-terminus of IRF3. Finally, we have shown that VZV inhibits the activation of NF-κB and that this inhibition is independent on the viral kinase ORF47p. In conclusion, we propose a model in which VZV interferes with the activation of IRF3 and subsequent induction of the antiviral response through its kinase ORF47p.

Collectively, our data support a mechanism by which VZV interferes with the activation of IRF3 and the subsequent induction of the antiviral response, which is summarized in the Figure 8. Through its kinase ORF47p, VZV induces an atypical phosphorylation of IRF3 that prevents its homodimerization and subsequent induction of target genes such as IFN-β and ISG15, thereby interfering with the activation of an antiviral state in the host cell.

## Materials and Methods

### Cell culture

Human embryonic kidney-293 (HEK-293) cell line (#ACC305, Deutsche Sammlung Von Mikroorganismen und Zellkulturen (DSMZ), Germany), Human embryonic kidney-293 expressing Toll-like receptor 3 (HEK-293-TLR3) cell line (#293-htlr3, InvivoGen) and Raw 264.7 macrophages (#TIB-71; ATCC, USA) were cultured in Dulbecco's Modified Eagle's medium (Lonza, Petit-Rechain, Belgium) supplemented with 10% fetal bovine serum, 1% glutamine and 1% non essential amino acids (Gibco®, Invitrogen). Human melanoma cell line (MeWo) (#93082609; ECACC, England) was cultured in Eagle's minimal essential medium (Lonza) supplemented with 10% fetal bovine serum and 1% glutamine.

### VZV infection

The VZV wild-type NIK strain is a gift from Dr. Arjen F. Nikkels. VZV ROka47S and VZV ROka66S were generated by insertion of Stop codons in the ORF47 and ORF66 and obtained form Jeffrey I. Cohen [21], [22]. The VZV ROka vaccine strain was obtained from Dr. Marvin Sommer [60]. Viruses were grown in human melanoma MeWo cell line. HEK-293 cells were infected with VZV by co-cultured with VZV-infected MeWo cells at a ratio 1 to 1, for 30 min at 37°C. The inoculum has been washed away and HEK-293 cells were cultured in fresh medium and harvested at various time points after infection.

### Isolation of peripheral blood mononuclear cells and generation of mature Myeloid DCs

Human peripheral blood was obtained from the “*Centre de Transfusion Sanguine*” (*Croix Rouge de Liège, Belgium*). PBMCs were isolated from buffy coat by density gradient sedimentation with Lymphoprep^TM^ (Axis-Shield, Norway). CD14 positive monocytes within PBMC were positively selected by incubating cells with anti-CD14 microbeads (Miltenyi, Germany) and running through a MS column (Miltenyi) in a magnetic field. The bound monocytes were then washed away from the column and culture at 37°C in 5% CO_2_ in RPMI1640 (Lonza) supplemented with 1% non essential amino acids, 1% sodium pyruvate 1% glutamine, 1% penicillin/streptomycin, 50 U/ml of IL-4 (Immunotools, Germany) and 800 U/ml of Granulocyte-Macrophage-Colony-Stimulating Factor (GM-CSF) (Immunotools, Germany). IL-4 and GM-CSF were added to the culture medium every two days for 7 days. On day 8, immature myeloid DCs were then treated with LPS (100 ng/ml) for 24 hours. On day 9, DCs phenotype was determined using flow cytometry. The cells consisted of >90% mature myeloid DCs as determined by their morphology and their cell surface phenotype (i.e CD1a^+^, CD80^+^ and CD86^+^).

### Flow cytometry analyzes and antibodies

Monoclonal antibodies specific for human CD1a (clone NA1/34; FITC conjugated), human CD14 (clone Tük4; RPE conjugated), human HLA DR (clone AB3; FITC conjugated) were purchased from Zebra Bioscience (Enschede, Netherlands). Monoclonal antibody for human CD86 (clone 2331; APC conjugated) was obtained from BD Pharmingen (Becton Dickinson, Erembodegem, Belgium). Monoclonal antibody for human CD80 (PE conjugated) was purchased from Immunosource (Halle-Zoersel, Belgium).

### VZV infection of mature myeloid DCs

MeWo cells seeded in 6-well plates were infected or not with VZV WT, VZV ROka47S and VZV ROka66S. 48 hours post-infection (when cells showed ∼80% of infectivity), 2×10^5^ mature DCs were added to each well of VZV-infected MeWo cells in RPMI supplemented with 1% non essential amino acids, 1% sodium pyruvate, 1% glutamine and 1% penicillin/streptomycin. After 24 h, the nonadherent DCs were removed and placed into new 6-well plates and cultured for supplemental 8 and 24 h, before being harvested as described previously [23].

### Reagents

LPS (#L4391) and Polyinosinic–polycytidylic acid potassium salt [poly (I:C)] (#P9582) were obtained from Sigma-Aldrich and used at a concentration of 400 ng/ml and 50 µg/ml, respectively. TNFα (#300-01A) was obtained from PeproTech EC (UK) and used at a concentration of 500 U/ml.

### Plasmids

ORF47-HA-tagged was kindly provided by Paul R. Kinchington. To construct IRF3 in frame with V5 epitope, IRF3 gene was amplified by PCR from the pEGFPC1-IRF3 (kindly provided by Dr. Denis Gerlier) and cloned into the pCDNA3.1/V5-His-TOPO (Invitrogen), according to the manufacturer's instructions.

### Cell transfection

For transient transfection experiments, HEK-293 cells were cultured at 60% confluence in 6-well plates. Then, the cells were transfected or not with the indicated vector using Transfectin (Bio-Rad Laboratories, Inc). Transfectin and DNA were mixed in OPTI-MEM® (Gibco®, Invitrogen) at a ratio 1.5 to 1. 24 hours later, cells were mock- or VZV-infected and harvested at the indicated periods of time.

### siRNA transfection

siRNA directed against TBK1 was synthesized by Eurogentec (Belgium) and the following sense sequence was used: 5′-CUCUUGGUAUGAAGAAAUU-3′. The Firefly Luciferase (GL2) siRNA (#4627, Ambion) was used as negative control. HEK-293 cells were seeded in 6-well plates and transfected with 100 pmol of the siRNA using the ProFection Mammalian Transfection System – Calcium Phosphate (#E1200, Promega) according to the manufacturer's instruction. After 24 hours, cells were non-infected, mock-infected or infected with the WT VZV. Twenty four hours post-infection, cells were harvested and IRF3 up-shift was monitored by SDS-PAGE. TBK1 knockdown was confirmed by Western Blotting.

### Lambda (λ) phosphatase treatment

λ-phosphatase was purchased from Westburg (#P0753). 40 µg of total cell extracts were incubated with 400 units of λ-Phosphatase in Reaction Buffer supplemented with MnCl_2_ at 30°C for 45 min. Dephosphorylated protein extracts were then incubated with SDS-loading buffer, boiled for two minutes and resolved by SDS-PAGE.

### Antibodies

The following antibodies were used in immunoblotting studies: anti-IRF3 (#550428, BD Biosciences, Belgium), anti-TBK1 (#IMG-270A, Imgenex-Bio-Connect BV, Netherlands), anti-TBK1 (#3013, Cell Signaling-Bioké, Netherlands), anti-HSP60 (#SPA-806, Stressgen-Gentaur Molecular Products, Belgium), anti-HA (#MMS-101R, Covance Europe), anti-β-actin (#A4700, Sigma, Bornem, Belgium). VZV anti-IE63 antibody (9D12) and anti-gE (VL8) have been described previously [61], [62]. VZV anti-ORF29p antibody was kindly provided by R. J. Cohrs. HRP secondary antibodies were obtained from Dako N. V. (Heverlee, Belgium).

### Western Blotting

Protein samples were incubated with sodium dodecylsulfate (SDS)-loading buffer (10 mM Tris-HCl pH 6.8; 1% SDS; 25% glycerol; 0,1 mM β-Mercaptoethanol, 0,03% bromophenol blue) and boiled for 2 min. Proteins were then separated by SDS-PAGE and transferred onto a polyvinylidene fluoride (PVDF) membrane (Roche Applied Sciences, Basel, Switzerland). The membrane was blocked with PBS-Tween containing 5% of non fat dry milk and then incubated with the indicated antibodies. The membrane has been developed using the Amersham ECL^TM^ Western Blotting Detection Reagents Kit (GE Healthcare UK, Buckinghamshire United Kingdom). Quantification was achieved by quantifying the densities of the bands obtained by Western Blotting, using the program Quantity One (Bio-Rad Laboratories Inc.).

### Co-immunoprecipitation experiments

HEK-293 cells were transfected either with HA-tagged ORF47 or V5-tagged IRF3 or co-transfected with both plasmids. 24 hours later, cells were infected or not with VZV ROka47S for another 24 hours. Cells were collected and total lysates were performed as described previously [40]. Either IRF3 or HA-ORF47 was immunoprecipitated from total extracts with anti-IRF3 (#sc-9082, Tebu-bio) and anti-HA (#MMS-101R, Covance Europe) antibodies overnight at 4°C. Immune complexes were then incubated during 2 hours at 4°C on a rotator with 30 µl of washed protein A-Agarose beads (Pierce, USA). Immunoprecipitates were collected by centrifugation and washed five times in NP-40 Wash Buffer as described previously [40]. Immunoprecipitates were then eluted in SDS 2% at 37°C for 10 min, boiled in SDS-loading buffer and loaded on SDS-PAGE. Samples were transferred onto a PVDF membrane. The membrane was then blocked in PBS-tween containing 5% of non-fat dry milk and blotted with anti-IRF3 (#550428, BD Biosciences, Belgium) and anti-HA (#MMS-101R, Covance Europe) antibodies.

### Nuclear extraction

Cells were washed with PBS and collected by scraping and centrifuged at 20000×g for 30 sec. Nuclear and cytoplasmic fractionations were separated as described previously [63]. Briefly, nuclei were lysed using cellular extract buffer (50 mM Tris pH 8.0; 280 mM NaCl; 0.5% IGEPAL; 0.2 mM EDTA; 2 mM EGTA; 10% glycerol; 1 mM DTT; 25 mM βglycerophosphate; 1 mM NaF; 1 mM Na_3_VO_4_; 1 mM PMSF; Complete^TM^ (Roche)). Nuclear proteins were then used either in electrophoretic mobility shift assay or in Western Blotting.

### Native PAGE

Cells were washed with PBS and collected by scraping and centrifuged at 20000×g for 30 sec. Total proteins extracts were performed in 75 mM NaCl; 50 mM Tris-HCl pH 7.5; 1 mM EDTA; 1% NP-40; 1 mM DTT; 25 mM βglycerophosphate; 1 mM NaF; 1 mM Na_3_VO_4_; 1 mM PMSF; Complete^TM^. Samples were prepared in the native PAGE buffer (62.5 mM Tris-HCl pH 6.8; 0.5% NaDeoxycholate; 15% glycerol; 0.03% bromophenol blue) and loaded on a Ready Gel 4–15% Tris-HCl (Bio-Rad Laboratories Inc) and run as described previously [45]. Proteins were then transferred onto a PVDF membrane. The membrane was blocked with PBS-Tween containing 5% of non fat dry milk and incubated with anti-IRF3 antibody.

### *In vitro* Kinase Assay

Cells were washed, collected and subjected to a total cell lysis in 25 mM Hepes-KOH pH 7.5; 150 mM NaCl; 0.5% Triton X-100; 1 mM DTT; 25 mM βglycerolphosphate; 1 mM Na_3_VO_4_; Complete^TM^. 900 µg of total proteins were incubated in the immunoprecipitation buffer (25 mM Hepes-KOH pH 7.5; 150 mM NaCl; 0.5% Triton X-100; 1 mM DTT; 25 mM βglycerophosphate; 1 mM Na_3_VO_4_; Complete^TM^) with 5 µl of anti-TBK1 antibody (#3013, Cell Signaling-Bioké) overnight at 4°C on a rotator. Immune complexes were then incubated during 2 hours at 4°C on a rotator with 30 µl of washed protein A-Agarose beads (Pierce, USA). Immunoprecipitates were collected by centrifugation, washed three times in the immunoprecipitation buffer and two times in the kinase buffer (25 mM Hepes-KOH pH 7.5; 10 mM MgCl_2_; 1 mM DTT; 25 mM βglycerophosphate; 1 mM DTT; 1 mM Na_3_VO_4_). Immunoprecipitates were then incubated in 10 µl of kinase buffer supplemented with 1 µl (10 µCi, 37×10^7^ mBq/mmol) of [γ-^32^P] ATP (PerkinElmer, Belgium) and 1 µg of GST-IRF3 bearing the Carboxy-end of IRF3 at 30°C for 30 min. The reaction was stopped by the addition of 10 µl of SDS loading buffer. Proteins were boiled for 2 min and separated by SDS-PAGE. The gel was transferred onto a PVDF membrane and exposed to Fuji X-ray films at −80°C. The membrane was then blocked in PBS-tween containing 5% of non-fat dry milk and immunoblotted with TBK1 antibody (#IMG-270A, Imgenex-Bio-Connect).

### Quantitative Real Time-PCR

Total RNA was isolated from cells using TriPure Isolation Reagent (Roche, Mannheim, Germany) according to the manufacturer's instructions. The reverse transcription was performed with 1 µg of total RNA incubated one hour at 37°C in 15 µl of First Strand Buffer (Invitrogen, Merelbeke, Belgium); 10 mM dNTP (Invitrogen); 50 U RNaseOUT^TM^ (Invitrogen); 10 mM DTT (Invitrogen); 0,1 µg/µl Random Primers (Invitrogen) and 250 U M-MLV Reverse Transcriptase (Invitrogen). Two microliters of cDNA were then subjected to quantitative PCR in ABI Prism 7000 sequence detection system (Applied Biosystems, Warington, United Kingdom), using the SYBR green mix (Applied Biosystems). The following primers were used: *iκbα* FW (5′-CCAACCAGCCAGAAATTGCT-3′), RV (5′-TCTCGGAGCTCAGGATCACA-3′); *isg15* FW (5′-CATCTTTGCCAGTACAGGAGCT-3′), RV (5′-ACACCTGGAATTCGTTGCC-3′); *18S* FW (5′-AACTTTCGATGGTAGTCGCCG-3′), RV (5′-CCTTGGATGTGGTAGCCGTTT-3′) (Eurogentec, Belgium). Primers used for *ifn-β* were described previously [64]. Experiments were done at least in triplicate. Differences (n-fold) between samples were calculated using the standard-curve method and the 2-ΔCt method [65]. *P-values* were calculated using the Graphpad Quickcalcs software (*t test*, www.graphpad.com). *P-value* <0.05, statistically different; *P-value* >0.05 not statistically different.

### Electrophoretic Mobility Shift Assay

For NF-κB binding assays, a probe carrying the κB site of the human immunodeficiency virus (HIV) long terminal repeat (LTR) promoter was used. The gel shift experiment was performed as described previously [50]. The gel was vacuum dried and exposed to Fuji X-ray films overnight at −80°C.
